# Natural Dietary Pigments: Potential Mediators against Hepatic Damage Induced by Over-The-Counter Non-Steroidal Anti-Inflammatory and Analgesic Drugs

**DOI:** 10.3390/nu10020117

**Published:** 2018-01-24

**Authors:** Herson Antonio González-Ponce, Ana Rosa Rincón-Sánchez, Fernando Jaramillo-Juárez, Han Moshage

**Affiliations:** 1 Department of Gastroenterology and Hepatology, University Medical Center Groningen, University of Groningen, 9713GZ Groningen, The Netherlands; herson_qfbd@hotmail.com; 2 Department of Molecular Biology and Genomics, University Center of Health Sciences, Universidad de Guadalajara, Guadalajara 44340, Mexico; anarosarincon@yahoo.com.mx; 3 Department of Physiology and Pharmacology, Basic Science Center, Universidad Autónoma de Aguascalientes, Aguascalientes 20131, Mexico; jara@att.net.mx; 4 Department of Laboratory Medicine, University Medical Center Groningen, University of Groningen, 9713GZ Groningen, The Netherlands

**Keywords:** analgesics, liver, acute liver failure, oxidative stress, antioxidant capacity

## Abstract

Over-the-counter (OTC) analgesics are among the most widely prescribed and purchased drugs around the world. Most analgesics, including non-steroidal anti-inflammatory drugs (NSAIDs) and acetaminophen, are metabolized in the liver. The hepatocytes are responsible for drug metabolism and detoxification. Cytochrome P450 enzymes are phase I enzymes expressed mainly in hepatocytes and they account for ≈75% of the metabolism of clinically used drugs and other xenobiotics. These metabolic reactions eliminate potentially toxic compounds but, paradoxically, also result in the generation of toxic or carcinogenic metabolites. Cumulative or overdoses of OTC analgesic drugs can induce acute liver failure (ALF) either directly or indirectly after their biotransformation. ALF is the result of massive death of hepatocytes induced by oxidative stress. There is an increased interest in the use of natural dietary products as nutritional supplements and/or medications to prevent or cure many diseases. The therapeutic activity of natural products may be associated with their antioxidant capacity, although additional mechanisms may also play a role (e.g., anti-inflammatory actions). Dietary antioxidants such as flavonoids, betalains and carotenoids play a preventive role against OTC analgesics-induced ALF. In this review, we will summarize the pathobiology of OTC analgesic-induced ALF and the use of natural pigments in its prevention and therapy.

## 1. Introduction

Humans have always relied on nature for their basic needs. For thousands of years, plants and their derivatives have formed the basis of sophisticated traditional medicine and have been an invaluable source of bioactive compounds with therapeutic potential. They play an important role all over the world in the treatment and prevention of human diseases [1,2]. The first records of the use of plants in medicine are from Mesopotamia and date from about 2600 BC. Most of the plant derivatives reported and used by the Mesopotamians are still in use as antibiotics and anti-inflammatory treatments [3]. Plant organs such as roots, leaves, fruits, seeds, and other sub-products contain a vast array of biological activities related to the presence of many chemically-diverse components. Therefore, plants represent an enormous resource for many kinds of bioactive molecules with highly specific biological activities for different diseases [4]. Many of the pharmacological activities of natural products are related to the presence of bioactive compounds with excellent capacity to reduce oxidative stress [5,6,7,8]. Although bioactive components are not always essential for the normal development and/or reproduction of a plant, they may play an important role as protective agents against environmental factors and predators, thus enhancing their survival [9,10]. The potential beneficial health effects of these dietary constituents are highly dependent upon their uptake from natural sources, their metabolism and their disposition in target tissues and cells [11,12]. Thus, dietary phytochemicals are important in human nutrition, medicinal chemistry and drug development [13]. It has been estimated that 25–50% of marketed drugs are derived from natural products and almost 50% of novel FDA-approved drugs between 1981–2006 have a natural product origin [2,14]. Since bioactive components from natural sources have evolved through natural selection, they are often perceived as showing more “drug-likeness and biological friendliness than totally synthetic molecules” [15]. Because these compounds have been selected for optimal interactions with cellular macromolecules in plants, they are likely to induce highly specific biological actions in mammals making them good candidates for further drug development [16]. Therefore, these compounds have proven to be a rich source of novel compounds for biological studies and an essential source for drug discovery [13].

In recent years, the self-consumption of over-the-counter (OTC) drugs such as non-steroidal anti-inflammatory and analgesic drugs has rapidly increased. It now represents a serious health problem around the world due to the high rates of morbidity and mortality both from conscious and unconscious overdoses [17,18,19,20]. Most of these intoxications cause either acute liver damage or chronic gastrointestinal, cardiovascular and renal diseases, with severe oxidative stress as a cause or consequence [21,22]. In this review, we will discuss the importance of dietary plant pigments in human health and their use as a preventive or therapeutic modality in the treatment of OTC drugs-induced acute liver failure.

## 2. Over-The-Counter (OTC) Non-Steroidal Anti-Inflammatory and Analgesic Drugs

Pain in daily life is the most common complaint among patients seeking care in an emergency department [23]. It is also a common experience for adolescents who suffer frequently from headache, abdominal and musculoskeletal types of pain [24]. There are two main classes of drugs recognized by the FDA: prescription and non-prescription (OTC) drugs. OTC drugs can be purchased and self-administered without a prescription or guidance of a general practitioner [25]. OTC drugs are sold worldwide, although the regulatory systems differ between countries [26]. OTC medications are the most commonly purchased and used drugs in the United States for the control of pain in patients with arthritis, minor surgery, headache, dysmenorrhea, backache, strains and sprains [27]. Due to the limited health care access of patients in developing countries, these drugs are often used inappropriately, increasing the risk of adverse effects, acute intoxications, and deaths [28]. OTC medications can be classified according to the World Health Organization Anatomical Therapeutic Chemical (ATC) classification into ten categories: analgesics, laxatives, antithrombotic agents, antacids, cough and cold preparations, antihistamines, dermatologicals, throat preparations, nasal preparations and antidiarrheals [29]. These medications are normally safe when used properly, but when used for extended periods or at high doses the incidence of adverse effects increases [30]. The OTC non-steroidal anti-inflammatory drugs (NSAIDs) ibuprofen, naproxen, and aspirin, as well as the analgesic acetaminophen are the most frequently used medications. They are used by approximately 23% of the population around the world. OTC analgesics and NSAIDs are mainly used by elderly patients to relief pain and inflammation [31]. However, there is a lack of information about the potential toxicity or adverse drug interactions associated with the long-term use and misuse of OTC analgesics. Not all consumers realize that prolonged daily use and intake of high doses of single OTC analgesics or combinations dramatically increase the risk of toxicity or adverse drug events, particularly for the hepatic, gastrointestinal, cardiovascular and renal systems. In addition, patients may not be aware that common cough, cold, or flu medications may contain OTC analgesics again increasing the risk of toxicity [18,32]. It is well known that use of analgesics is critically relevant for public health, but there are no representative population-based data on their actual use [33]. The use and prescription of OTC analgesics in patients known to be at high risk to develop adverse effects has been regulated to reduce the incidence of intoxications and mortality [34,35].

### Epidemiology of OTC Non-Steroidal Anti-Inflammatory and Analgesic Drug (Over) Use 

Pain in the United States is one of the most important causes of lost labor productivity. In 1985, it was estimated that the total loss of work days due to pain equaled $55 billion [36]. Likewise, a significant percentage of the adult population in Canada lost work days because of the high prevalence of headaches, including migraine [37]. Because of the high incidence of pain, the use and sales of OTC analgesic drugs has steeply increased. A survey in UK demonstrated that 60% of OTC NSAIDs prescribed were for elderly patients. Thirty-eight percent were taking drugs that can interact with NSAIDs, 46% had one or more conditions that may be aggravated by NSAIDs, and 18% had side effects [38]. Most of these analgesics and NSAIDs are sold over-the-counter [39]. In the UK, acetaminophen (APAP) has been reported as the most commonly drug used for self-poisoning and overdoses with an increase of ≈28% from 1976 to 1990, and in 1993, 48% of all overdoses reported in the UK involved acetaminophen or acetaminophen-containing drugs (reviewed in [40]). A survey of medication use in the United States under participants over 18 years old demonstrated that 81% of them used at least one OTC or prescription drug in the preceding week, and the highest prevalence of medication use was observed in women over 65 years old. In addition, six of the ten most frequently used drugs are OTC drugs. The most frequently used drugs were acetaminophen, ibuprofen and aspirin [31]. An Emergency Department survey at the USA showed that 56.2% of 546 patients interviewed took OTC analgesics, including acetaminophen (53%), ibuprofen (34%), aspirin (17%) and naproxen (7.8%); and 6.2% of these 546 patients exceeded the manufacturer’s maximum recommended daily dose for at least one medication for at least one day during the three days preceding the evaluation [28]. A study in the Czech Republic from 2007 to 2011 to determine the toxicological characteristics of suicide attempts by deliberate self-poisoning reported that acetaminophen, diclofenac and ibuprofen were related to ≈16% of the cases from 2393 calls concerning children and adolescents and 30.3% of these cases were related to drug combinations including acetaminophen [41]. A Dutch survey performed in 2014 revealed that almost one-third of the general population used NSAIDs prior to the survey and 31% of those used two or more NSAIDs. In addition, 23% of NSAID users consumed these drugs for more than seven days and 9% of this population exceeded the daily maximum dose. These results suggest that at least 333,000 Dutch adults exceeded the maximum dose of OTC NSAIDs [35]. In Germany, analgesic use increased from 19.2% of the population in 1998 to 21.4% in the period 2008–2011. This increase is due exclusively to the increase in OTC analgesic use from 10.0% to 12.2%. Ibuprofen was the most commonly used analgesic. In the period 2008–2011, the use of analgesics was significantly higher in women than men (25.1% vs. 17.6%) with ibuprofen and acetaminophen being the most commonly consumed analgesics. From all the analgesic users, 4.9% (74/1490) used a combination of acetaminophen and ibuprofen. NSAIDs were used mostly in combination by 6.0% of the participants during the seven-day period [33]. A recent Swedish survey concluded that it is important to inform the population about the therapeutic use and risks of the consumption of OTC analgesics since there is a significant influence of parents and peers on the young population [26]. Due to the risk of unintentional overdoses with OTC medications, the prevalence of the problem and the frequent lack of an expert to guide and inform consumers on the proper use, a report in the USA concluded that OTC (over)consumption is a serious public health threat. This health issue requires urgent attention because many consumers are not able to identify or differentiate the active component(s) in OTC analgesic medications and their biological activities, nor do they adhere to the recommended intake instructions [42]. In addition, there is a high prevalence of drug–drug interactions resulting from the co-administration of NSAIDs and other commonly used medications by patients with osteoarthrosis (OA) and rheumatoid arthritis (RA). Therefore, it is necessary to maintain medical supervision of those patients with OA and RA receiving OTC NSAIDs and other medications, as well as to inform them about the risk of toxicity and how to identify toxicity [43].

In summary, when OTC analgesics are taken as recommended by the general practitioner, they are a safe, effective, and economical treatment to relief pain, inflammation, and fever. Nevertheless, because of their wide availability and perceived safety, OTC analgesics are frequently overconsumed resulting in their hepatic, gastrointestinal and cardiovascular side effects [32,44].

## 3. Liver Histology and Structures

**Figure 1:**
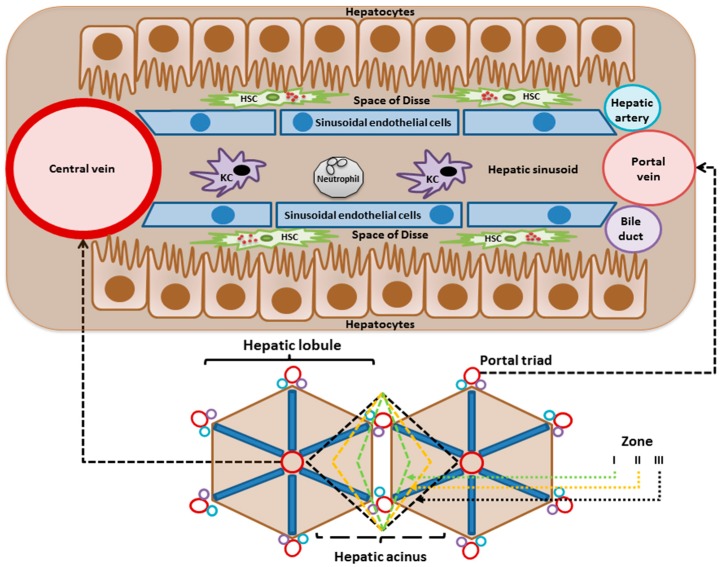
Structure and cell types of a normal liver. HSC, hepatic stellate cells; KC, Kupffer cells.

The liver is the main site for the biotransformation of exogenous chemical compounds (xenobiotics) consumed by humans such as drugs. Therefore, understanding drug toxicity is only possible with a thorough understanding of liver structure and function. The liver is a large, solid and highly vascularized organ with a pivotal function in metabolic homeostasis, detoxification, and immunity in the human body [45]. The liver has endocrine and exocrine properties. The main endocrine functions are the secretion of different hormones such as insulin-like growth factors, angiotensinogen, and thrombopoietin; and bile secretion as the major exocrine function [46]. The liver is capable to synthesize and degrade a wide variety of molecules in a regulated way such as carbohydrates, lipids, amino acids, bile acids and xenobiotics [47]. The hepatic parenchyma is organized in lobules, composed of functional units consisting of epithelial cells (hepatocytes) and non-parenchymal cells including sinusoidal endothelial cells, Kupffer cells, stellate cells, cholangiocytes (biliary epithelial cells) and immune cells (Figure 1) [48]. The anatomical features that characterize the liver architecture are: the portal triad (bile duct, hepatic artery, and portal vein), the central vein, and the hepatic sinusoids [47]. Hepatocytes form plates and are in contact with the blood since they are juxtaposed with hepatic sinusoids [49]. The hepatic sinusoid is a microvascular unit formed by endothelial cells distinguished by fenestrations and separated from the hepatocytes by the subendothelial space of Disse, where stellate cells reside. This mass of cells is pervaded by a system of secretory channels (bile ducts) which empty into the intestine (Figure 1) [50].

The hepatocytes produce many circulating plasma proteins such as albumin, coagulation factors, and acute phase proteins. Hepatocytes metabolize and store gut-derived nutrients and glycogen and generate glucose under conditions of starvation. Hepatocytes have a central role in the regulation of lipid metabolism and synthetize lipoproteins. Bile acids are de novo synthetized by the hepatocytes using cholesterol as precursor [51]. Hepatocytes can adapt to the metabolic needs in the body through regulation of protein synthesis and/or zonation. This is controlled by both hormonal and metabolic signals [52].

### 3.1. Drug Biotransformation

When a foreign compound (xenobiotic) enters the body, it is metabolized by members of a group of hepatic enzymes known as xenobiotic-metabolizing enzymes, which include phase I oxidative enzymes and phase II conjugating enzymes [53]. Hepatocytes carry out most of the metabolic functions of the liver. Both endobiotics and xenobiotics are metabolized across the liver cell plate and secreted into bile [47]. The portal vein brings blood to the liver from the splenic, superior mesenteric, inferior mesenteric, gastric, and cystic veins. Portal blood flow comprises 75–85% of hepatic blood supply, and the remaining 15–25% is delivered by the hepatic artery [54]. Therefore, all absorbed xenobiotics eventually reach the liver to be metabolized and excreted. Due to this first pass metabolism, the concentration of xenobiotics in the systemic circulation is low compared to the portal circulation [51,55]. Xenobiotics must be converted into polar (hydrophilic) metabolites to facilitate their excretion. These water-soluble conjugates can be excreted from the body through the kidneys [56]. The cytochrome P450 (CYP450) system is a phase I microsomal enzyme-family that participates in the metabolism of xenobiotics via oxidation, reduction or hydrolysis, yielding more polar metabolites whereas phase II metabolism via conjugation reactions like glucuronidation or sulfation, facilitates their excretion together with the phase III drug transporters.

The phase I enzymes belong mainly to the flavin-containing monooxygenase (FMO) superfamily and the CYP superfamily. These enzymes are important in the metabolism of most clinically used drugs, but they also participate in the metabolic activation of chemical carcinogens and toxins. The phase II enzymes conjugate the primary metabolites into more polar species, facilitating their excretion from the body. Therefore, the accumulation of (intermediary) metabolites is dependent on the relative expression of phase I and phase II enzymes. This, in turn, is dependent on the extent of induction of these enzymes and gene polymorphisms [57,58]. Although these reactions are meant to detoxify xenobiotics into less toxic metabolites, they can sometimes generate reactive intermediates (electrophilic metabolites) inducing cell toxicity [59]. The phase III drug/metabolite transporters such as P-glycoprotein (P-gp), multidrug resistant-associated proteins (MRPs) and organic anion transporting polypeptide 2 (OATP2) play an important role in the determination of the systemic bioavailability of many drugs since they are capable to reduce drug absorption, prevent their access to the systemic circulation and enhance their excretion to the gut lumen. The induction of these transporters is regulated by the activation of several nuclear transcription factors like the orphan nuclear receptors such as pregnane X receptor (PXR), farnesoid X receptor (FXR) and constitutive androstane receptor (CAR). Thus, the activation or induction of phase I and II enzymes and phase III transporters provide an important way to protect the body from xenobiotics and other cellular stressors (reviewed in [60]).

It has been demonstrated that the expression of CYP450 enzymes is higher in the pericentral area than in the periportal area, and that pericentral hepatocytes have a larger area of smooth endoplasmic reticulum and a higher surface density of CYP450 compared to periportal hepatocytes [61]. Thus, the pericentral area is more susceptible to toxic metabolites generated by CYP enzymes.

Since most of the CYPs are inducible, hepatic drug metabolism is regulated at the level of gene expression. However, posttranslational modifications as well as, e.g., alterations in blood flow, also contribute to the regulation of drug-metabolizing activity [56].

In addition, the expression of enzymes and xenobiotic transporters may be regulated through the activation of specific receptors by xenobiotics [53].

### 3.2. Free Radicals and Reactive Oxygen Species

Production of energy, ATP, in cells requires oxygen consumption. Free radicals are produced as a result of aerobic ATP production via the mitochondrial electron transport chain [62,63,64].

Harman proposed the “free-radical theory” of ageing in the mid-1950s, suggesting that endogenous reactive oxygen species, produced by the metabolism of mammalian cells, induce cumulative damage [65]. This concept was initially controversial until superoxide dismutase (SOD) was identified [66]. SOD is an enzyme that inactivates superoxide anions produced by the aerobic metabolism of cells, providing a mechanistic link to support Harman’s hypothesis. Ageing and the development of age-related diseases appears to be a consequence of increased levels of intracellular oxidants that induce significant effects such as the activation of signaling pathways and the damage of cellular components [67].

Reactive oxygen species and reactive nitrogen species (ROS and RNS, respectively) are fundamental in modulating mitochondrial functions via the regulation of electron transfer chain enzymes and mitochondrial membrane potential [68]. ROS are crucial for various cellular processes, including cell proliferation [69], apoptosis [70,71], cytotoxicity against bacteria and other pathogens [72], cell adhesion and immune responses [73]. ROS and RNS also act as second messengers in redox signaling [74].

Mitochondrial metabolism, although essential for cellular homeostasis, is also considered the main source of intracellular ROS: superoxide radicals are mainly generated by complex I (NADH:ubiquinone oxidoreductase) and complex III (ubiquinol-cytochrome *c* reductase) of the electron transport chain [75]. However, mitochondrial metabolism is not the only source of oxidants. Under physiological conditions, cytosolic enzyme systems including NAPDH oxidases (NOX), microsomal monooxygenases (cytochromes P450), xanthine oxidase (XO), nitric oxide synthases (NOS), lipoxygenases (LOX), cyclooxygenases (COX) and myeloperoxidases can also produce ROS and RNS [67,76,77,78]. ROS and RNS are generated in excess in some pathological conditions such as neurodegenerative disorders, cancer, diabetes and cardiovascular and liver diseases, and cause cell damage due to their high reactivity with cellular biomolecules [79].

ROS and RNS comprise a group of different molecules, including free radicals such as superoxide anion (O_2_^•−^), hydroxyl radical (^•^OH) and nitric oxide (NO^•^), and non-radicals, such as hydrogen peroxide (H_2_O_2_), singlet oxygen (^1^O_2_) and peroxynitrite (ONOO^−^). Many free radicals are extremely unstable, whereas others are freely diffusible and relatively long-lived [64,79].

Additional endogenous non-mitochondrial sources of free radicals include Fenton’s reaction, peroxisomal beta-oxidation, and the respiratory burst of phagocytic cells [22,80]. In addition, the production of pro-inflammatory cytokines by activated macrophages and neutrophils and viral proteins stimulate the generation of ROS [81]. The auto-oxidation of many biologically important molecules and the electron delocalization that takes place in reactions of heme-containing proteins, also results in the production of oxidants [64]. The most relevant exogenous sources of free radical production are pollutants/toxins such as cigarette smoke, alcohol, ionizing and UV radiation, pesticides, and ozone [22]. Moreover, several OTC anti-inflammatory and analgesic drugs induce excess generation of ROS and RNS when used at high or prolonged doses due to their metabolism in the liver, e.g., acetaminophen [82,83], diclofenac [84,85], aspirin [86], and ibuprofen [87].

### 3.3. Cellular Oxidative Stress

**Figure 2:**
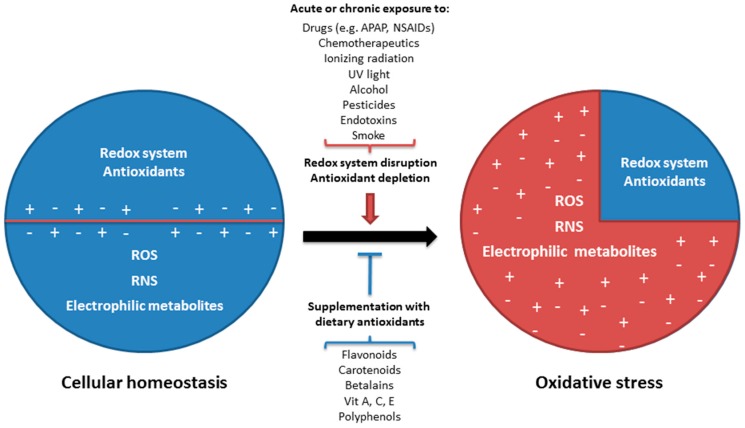
Disruption of the redox system leads to oxidative stress and cellular injury. ROS, reactive oxygen species; RNS, reactive nitrogen species; APAP, acetaminophen; NSAIDs, non-steroidal anti-inflammatory drugs.

The excessive generation of ROS and RNS cause damage to cellular macromolecules such as nuclear and mitochondrial DNA, RNA, lipids and proteins by nitration, oxidation, and halogenation reactions, leading to impaired cellular functions and increased mutagenesis [88,89]. Oxidative damage to essential cellular components (macromolecules, organelles) is generally considered as an important mechanism in the pathophysiology of inflammatory diseases [90]. Oxidative stress is the result of either increased generation of ROS and RNS by endogenous and/or exogenous factors, or the result of a decline of the cellular antioxidant capacity (Figure 2) [91].

Cells are protected against ROS and RNS by both enzymatic and non-enzymatic antioxidant defense systems [92,93]. Excessive generation of oxidants might saturate the antioxidant pathways leading to cellular injury. Thus, free radicals react with membrane phospholipids or lipids from dietary intake, inducing lipid peroxidation (LPO) and the generation of highly toxic products such as trans-4-hydroxy-2-nonenal (4-HNE), 4-hydroperoxy-2-nonenal (HPNE) and malondialdehyde (MDA). 4-HNE, HPNE and MDA can subsequently react with DNA bases such as deoxyadenosine, deoxycytidine and deoxyguanine to form various mutagenic exocyclic adducts implicated in, e.g., hepatocarcinogenesis [94,95]. ROS, RNS and LPO products can also induce expression of genes implicated in the inflammatory response and the pathogenesis of several diseases such as the transcription factor Nuclear Factor NF-κβ, iNOS, and cyclooxygenase-2 (COX-2) [79].

Protein carbonylation is not only associated with an age-related diminished capacity of the antioxidant defense systems, but also with increased generation of ROS and RNS. Protein carbonylation results in altered protein structure and function [96].

Oxidative stress also induces a cellular stress response through the activation of the main stress signaling pathways such as the extracellular signal-regulated kinase (ERK), c-Jun N-terminal protein kinase (JNK) and p38 mitogen-activated protein kinase (MAPK) signaling cascades, the phosphoinositide 3-kinase (PI(3)K)/Akt pathway, the nuclear factor NF-κB signaling system, p53 pathway, and the heat shock response [67]. Some of these pathways can also be activated through other mechanisms like DNA damage [97,98] and stimulation of growth-factor receptors [99,100].

In addition, the complex formed by the redox regulatory protein thioredoxin (Trx) and the apoptosis signal-regulating kinase (ASK1) can be dissociated by oxidative stress and induce the subsequent activation of the JNK and p38 kinases [101]. Glutathione S-transferase binds to JNK to keep it inactivated under normal conditions, but under oxidative stress conditions this interaction can be disrupted [102]. These results suggest that there is a link between alterations in the intracellular redox system and the activity of stress-activated pathways [67].

## 4. Drug-Induced Liver Injury

Drug-induced oxidative stress is a frequent cause of hepatotoxicity, liver injury and failure. In this regard, drug-induced oxidative stress is considered an important event that can lead to the initiation or progression of liver injury [103]. It is frequently accompanied by clinical signs of acute hepatitis and/or cholestasis [104]. Drug-induced liver injury (DILI) accounts for almost 50% of the cases of acute liver failure (ALF) in the United States [105,106], and for more than 50% in UK. Several factors have been identified that predict drug-induced liver injury (DILI), e.g., dose, alcohol consumption, use of concomitant drugs, nature of the drug, time of exposure, age, preconditioning diseases, and congenital anomalies [104,107]. DILI may be classified as non-idiosyncratic or idiosyncratic. Idiosyncratic drug reactions are unpredictable and independent, and can occur from intermediate to long periods of exposure by an activation of the immune response, inflammation, and cell death (mostly apoptosis). Drug-induced predictable liver injury, such as from acetaminophen, can occur within few hours or days and are mediated by the production of free radicals or electrophilic metabolites from drug biotransformation inducing organelle stress and cell death (both necrosis and apoptosis) [104,108,109]. Necrosis involves the depletion or inactivation of endogenous antioxidants and the induction of cellular stress including mitochondrial stress and decreased ATP synthesis which leads to cellular dysfunction and ATP-independent death. In contrast, apoptosis is an ATP-dependent mechanism involving activation of nucleases [108,110]. Therefore, the activation of death-signaling pathways such as JNK is an important event in DILI [111].

The liver plays a critical role in the disposition of orally administered therapeutic agents. It is the port-of-entry of most orally taken drugs and it represents the major site of drug biotransformation making the liver susceptible to drug-induced toxicity. Products of drug biotransformation (electrophilic compounds and free radicals) have been implicated as causative agents of liver toxicity through direct injury to the hepatocytes by interfering with critical cellular functions (e.g., ATP production), modifying important biomolecules (e.g., proteins, lipids, or nucleic acids), depleting cellular antioxidants, inducing cellular oxidative stress [112,113].

Cellular functions can be affected by both direct effects on organelles (e.g., the mitochondria, the endoplasmic reticulum, the cytoskeleton, microtubules, or the nucleus), and indirectly modulating signaling kinases, transcription factors, and gene expression. These cellular effects can activate the immune response via the release of pro-inflammatory cytokines and/or cell debris into the blood-stream, resulting in the recruitment of immune cells (neutrophils), cellular stress and hepatocyte death that ultimately induce liver injury and failure [56,114,115,116].

Mitochondria play an important role in the development of DILI since they are an important regulator of cellular homeostasis and their dysfunction can trigger liver cell toxicity resulting in mild to fulminant hepatic failure [117]. Very often, cell death is associated with depletion of mitochondrial glutathione and not with loss of cytoplasmic glutathione [118]. Therefore, it is important to elucidate whether drug metabolites have direct effects on mitochondrial function (e.g., via inhibition of electron transport chain or increasing lipid peroxidation and membrane permeability) resulting in hepatocellular death or indirect effects via activation of the mitochondrial pathways of programmed cell death [119].

### 4.1. OTC Non-Steroidal Anti-Inflammatory and Analgesic Drugs-Induced Acute Liver Injury

NSAIDs are the most widely used OTC drugs as well as the most prescribed class of drugs for a variety of conditions [120,121]. The group of NSAIDs is composed of a large class of chemical compounds with the same biological activity: blocking the production of prostaglandins (PGs) through the inhibition of the enzyme cyclooxygenase (COX). The COX enzyme is present as two isoforms, each with distinct functions. COX-1 is an isoenzyme constitutively expressed in the stomach, kidney, intestinal mucosa, and other tissues, and is involved in the biosynthesis of PGs serving homeostatic functions. It plays an important role in vasoconstriction and platelet aggregation. 

**Figure 3:**
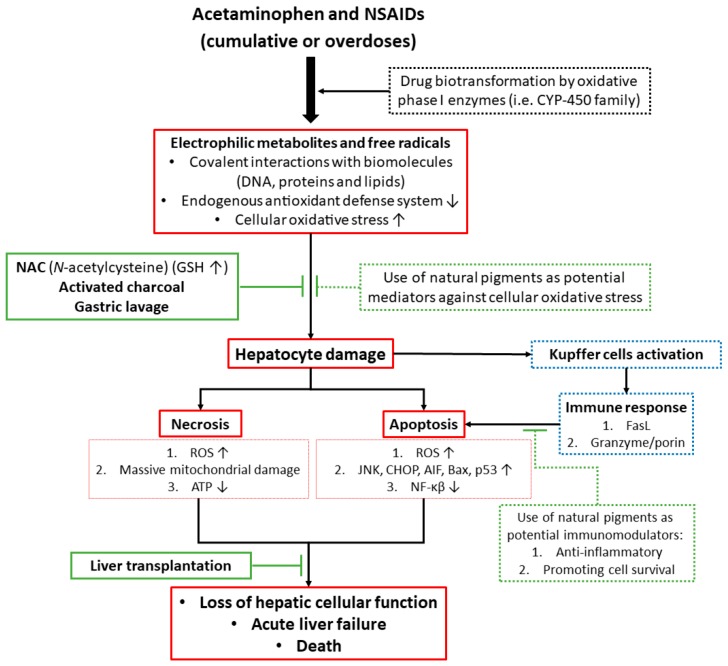
Biotransformation of drugs by oxidase enzymes triggers an intracellular chain reaction mediated by the overproduction of reactive metabolites and free radicals which leads to cell death. Current treatments for drug-induced liver injury are limited. Natural pigments represent a potential alternative treatment to prevent acute liver failure. CYP450, cytochrome P450; GSH, glutathione; ROS, reactive oxygen species; JNK, c-Jun N-terminal protein kinase; CHOP, C/EBP homologous protein; AIF, apoptosis-inducing factor; Bax, bcl-2-associated X protein.

The inducible isoenzyme COX-2 is induced during inflammation, where it causes vasodilation, and other pathologic conditions [122,123]. Acetaminophen and NSAIDs misuse or overdoses have potential significant adverse effects that include gastrointestinal ulcers with consequential bleeding, renal dysfunction, and hepatotoxicity, as well as the risk of death (Table 1) [120,124]. In fact, after antibiotics and anticonvulsants, NSAIDs are considered the most common medications associated with drug-induced liver injury mainly through an idiosyncratic form of hepatotoxicity [125,126]. NSAIDs have also been associated with increased ROS production and oxidative stress. Excessive ROS generation and disturbed cellular redox balance are considered to be important factors in the dysfunction of various biological signaling pathways (Figure 3) [121,127,128].

Current treatments for (drug-induced) acute liver failure are limited since patients usually present at a late stage. Early diagnosis improves prognosis. Treatment with *N*-acetylcysteine (NAC) is the only clinically used antidote against acetaminophen intoxication. NAC is a precursor of glutathione (GSH) and reduces oxidative stress but is not always effective and liver transplantation is often required. 

Activated charcoal is another potential treatment to reduce the absorption of NSAIDs and liver damage but is only effective within the first few hours after intoxication (Table 1). Therefore, alternatives for the treatment of DILI are needed. Natural pigments with antioxidant and therapeutic activity from plants, fruits and their derivatives might be used as an alternative strategy to reduce the incidence and effects of DILI. The use of antioxidants from natural and dietary sources represents a rational defense to prevent or cure liver diseases related to cellular oxidative stress. Promising results of natural antioxidant compounds against different types of liver toxicity or diseases have been obtained in cell culture models and animal studies, but their efficacy in clinical studies remains uncertain (Figure 3) [103,129].

Crofford [123] described that approximately 15% of patients taking NSAIDs display increased markers of liver injury such as alanine aminotransferase (ALT) and aspartate aminotransferase (AST). Dose reduction or discontinuation of the drug can normalize these markers. NSAIDs are generally grouped according to their chemical structures, plasma half-life, and COX-1 versus COX-2 selectivity. Structurally, NSAIDs include several groups such as salicylic acids, acetic acids, propionic acids, fenamic acids, pyrazolones, oxicams, sulfonamide, sulfonylurea, as well as non-acidic drugs. In this review, we will focus on liver injury induced by misuse or overdoses of acetaminophen, acetylsalicylic acid, diclofenac, naproxen, and ibuprofen.

#### 4.1.1. Acetaminophen (APAP, Paracetamol)

Acetaminophen (APAP) is the main cause of drug intoxication and acute liver failure (ALF) worldwide. This is due primarily because of its perceived safety. Acute hepatotoxicity may be induced by a single overdose or unexpected side-effects (idiosyncratic) [130,131]. Toxicity by APAP accumulation is also common in frequent users. In fact, some patients frequently ingest different acetaminophen-containing products at the same time, generating overdoses and toxicity [132]. Thus, acetaminophen is a classical dose-dependent hepatotoxin that is responsible for almost 50% of all ALF cases in many Western countries [133,134].

At therapeutic doses, APAP is mainly metabolized by microsomal enzymes in the liver and eliminated for approximately 85–90% via glucuronidation and sulfation reactions [135]. However, at high doses (or patients with risk factors as chronic alcohol ingestion and malnutrition), the conjugation pathways are saturated, and part of the drug is converted by the CYP450 drug metabolizing system (mainly CYP1A2, CYP2E1 and CYP3A4) to the highly reactive metabolite *N*-acetyl-*p*-benzoquinone imine (NAPQI) that reacts with protein sulfhydryl groups of cysteine. Once generated, NAPQI is immediately inactivated by endogenous reduced glutathione (GSH) to form NAPQI-GSH conjugates which are excreted through the urine. However, when the hepatic reservoir of GSH is depleted, cellular organelles (e.g., mitochondria and endoplasmic reticulum) are exposed to the highly reactive metabolite NAPQI. NAPQI reacts with (membrane) biomolecules forming adducts and resulting in the disruption of cellular homeostasis [7,81,136,137].

Activation of the JNK pathway plays an important role in APAP-induced liver injury and hepatocyte death. Models of genetic JNK knock-out and hepatoprotective compounds with JNK suppressing activity may prevent APAP-induced oxidative stress, cell death and liver failure. Once activated, JNK translocates to the mitochondria inducing mitochondrial permeability transition (MPT), mitochondrial dysfunction and cell death [118]. Protein kinase C (PKC) may also play a critical role in APAP-induced hepatotoxicity via the JNK signaling pathway since treatment with PKC inhibitors (Ro-31-8245, Go6983) protected primary mouse hepatocytes. Ro-31-8245 treatment increased p-AMPK levels (phosphorylated AMP-activated kinase), and promoted autophagy. Treatment with the PKC inhibitor Go6976 inhibits JNK activation and translocation, protecting hepatocytes against APAP cytotoxicity [138].

In mouse models and in human hepatocytes, APAP-induced liver injury involves mitochondrial damage, oxidative stress, JNK activation, and nuclear DNA fragmentation. Thus, protein adducts in mitochondria damage the electron transport chain, increase oxidative stress and disturb the innate immune system of the liver [139,140]. Of note, liver injury is aggravated by subsequent oxidant stress via ROS. The enhanced superoxide formation leads to generation of the potent oxidant peroxynitrite in mitochondria [141].

In the mouse model, APAP toxicity produces very early activation (phosphorylation) of JNK in the cytoplasm. Activated JNK translocates to the mitochondria and increases oxidant stress. This causes the formation of the mitochondrial permeability transition pore and collapse of the mitochondrial membrane potential, as well as a drop in ATP production [140,142,143]. In addition to depleting intracellular GSH, APAP treatment also increases lipid peroxidation and causes hepatic DNA fragmentation. The combination of massive mitochondrial dysfunction and nuclear disintegration leads to cellular necrosis [144,145,146]. Alterations in hepatic innate immunity and inflammation also play a significant role in the progression of hepatic failure after acetaminophen overdose [135,147].

The primary mechanism of cell death in acetaminophen-induced liver failure is thought to be necrosis, however, some reports have shown that apoptosis may also play a significant role [140]. In the human hepatoblastoma cell line (HuH7), activation of caspases was observed and the manifestation of apoptosis was preceded by a translocation of cytochrome c from mitochondria to the cytosol [148]. It remains to be elucidated whether hepatoma cell lines accurately reflect the in vivo metabolism of APAP. Recent studies also demonstrated increased serum markers of apoptosis, such as caspase-cleaved cytokeratin-18 (M30), in the early phase of acetaminophen-induced ALF in humans [149]. The exact mechanism(s) and optimal management of APAP-induced acute liver failure still need to be clearly elucidated to reduce the high morbidity and mortality of APAP-induced ALF [150].

#### 4.1.2. Acetylsalicylic Acid (ASA, Aspirin)

Acetylsalicylic acid (ASA) is a widely used NSAID due to its pharmacological properties (including analgesic, antipyretic, anti-inflammatory and anti-platelets effects), as well as its easy availability. ASA is an irreversible inhibitor of both cyclooxygenase isoenzymes, COX-1 and COX-2. After oral administration, ASA is absorbed from the stomach and small intestine, primarily by passive diffusion across the gastrointestinal tract [151]. ASA is rapidly deacetylated to salicylic acid by esterases in the gastrointestinal mucosa, in the blood and in the liver. The oxidation of salicylic acid in human liver microsomes produces two metabolites, 2,5-dihydroxybenzoic acid (gentisic acid) and 2,3-dihydroxybenzoic acid. The major human cytochrome P450 involved in both biotransformation reactions is CYP2E1 [152].

Liver toxicity induced by ASA is considered to be dose-dependent, although predisposing conditions may exist that increase the individual risk of liver damage [153,154]. Oxidative stress is one of the mechanisms associated with the adverse effects of ASA and salicylic acid may induce cytochrome P450-mediated lipid peroxidation in liver microsomes [155]. On the other hand, antioxidant properties of this drug have also been reported [156]. Doi and Horie [86] also analyzed mitochondrial dysfunction and oxidative stress in salicylic acid-induced liver injury. In rat hepatocytes, salicylic acid significantly increased the leakage of lactate dehydrogenase and increased thiobarbituric acid reactive substances (TBARS) formation, a marker of lipid peroxidation, whereas antioxidants (promethazine and DPPD (*N*,*N*′-diphenyl-p-phenylenediamine)) suppressed both harmful effects. TBARS formation in rat liver microsomes was also suppressed by diethyldithiocarbamate (a specific inhibitor of CYP2E1) and diclofenac (a specific inhibitor of CYP2C11). Salicylic acid also significantly decreased ATP content in isolated rat hepatocytes and mitochondrial respiration. The authors suggest that salicylic acid impairs mitochondrial function leading to lethal liver cell injury by lipid peroxidation.

Raza et al. [157] analyzed the oxidative effects of ASA in cultured human hepatoma cells (HepG2) and reported a cascade of adverse events starting with the overproduction of cellular ROS through the uncoupling of the complex I (NADH:ubiquinone oxidoreductase) and IV (cytochrome c oxidase) of the mitochondrial electron transport chain and ultimately resulting in reduced levels of GSH. The altered MPT disrupted the mitochondrial ATP synthesis, decreased the expression of the anti-apoptotic protein Bcl-2 and induced the activation and release of pro-apoptotic proteins to induce cell death. ASA-induced cytotoxicity was augmented by inhibition of GSH synthesis and attenuated by increasing the GSH pool. The authors conclude that ASA-induced toxicity in human HepG2 cells is mediated by increased metabolic and oxidative stress, accompanied by mitochondrial dysfunction, resulting in apoptosis [158].

Tassone et al. [159] reported a pilot study in 22 newly diagnosed diabetic patients treated with ASA (100 mg/daily for four weeks). The authors suggest that ASA treatment for primary prevention in diabetic patients causes oxidative stress and impairs vascular function.

In addition to mitochondrial dysfunction, ASA treatment can also lead to accumulation of free fatty acids in the liver leading to massive hepatic steatosis [154]. The mechanism of ASA-induced hepatotoxicity is different from that of other NSAIDs. As described above, ASA is first hydrolyzed by non-specific esterases into salicylic acid. In mitochondria, salicylic acid may form salicylyl-coenzyme A (CoA) conjugates, thus sequestering extramitochondrial CoA. This conjugate indirectly inhibits β-oxidation of long-chain fatty acids since CoA is necessary to transport free fatty acids into the mitochondria [160,161]. The inhibition of mitochondrial β-oxidation of long-chain fatty acids by ASA may lead to microvesicular steatosis known as Reye’s syndrome [113,162]. Salicylic acid can also inhibit Krebs cycle enzymes such as α-ketoglutarate dehydrogenase and succinate dehydrogenase leading to mitochondrial dysfunction [162].

Finally, Jain et al. [151] published that treatment of female rats with ASA (100 mg/kg b.w. (body weight)) caused significant histopathological alterations in the liver, including degenerative and pyknotic changes in the nuclei, vacuolization and clear dilatations in the sinusoids and hypertrophy of hepatocytes.

#### 4.1.3. Diclofenac

Diclofenac is a commonly prescribed NSAID of the phenyl-acetic acid class. Diclofenac has been used in a variety of inflammatory conditions and has strong anti-inflammatory activity, although analgesic and antipyretic properties have also been reported [163]. In contrast to traditional NSAIDs, diclofenac appears to have a higher selectivity for COX-2 than COX-1. Diclofenac has been associated with serious dose-dependent gastrointestinal, cardiovascular, and renal toxicity [164]. Diclofenac-induced liver injury has been used as a model of drug-related toxicity. The hepatotoxicity induced by diclofenac is mainly due to its metabolites, but genetic factors can also increase the susceptibility to produce and accumulate the reactive acylglucuronide metabolite which triggers an immune response and liver injury [84,154,165,166,167]. The bioactivation of diclofenac by CYP2C9 or CYP3A4 yields thiol-reactive quinone-imines which in turn are conjugated by UDP (Uridine 5′-diphospho)-glucuronosyltransferase (UGT2B7) into protein-reactive acyl-glucuronides. Therefore, both disruption of mitochondrial function and alterations in the redox state due to oxidative or nitrosative stress appear to be the main mechanisms of diclofenac-induced cell death and liver injury.

Laine et al. [168] conducted a long-term prospective clinical trial to analyze the frequency of diclofenac-induced adverse hepatic effects. A total of 17,289 arthritis patients received diclofenac for a mean duration of 18 months. Increased serum transaminase occurred primarily within the first 4–6 months of therapy and was observed in 3.1% of arthritic patients. Of note, ALT/AST ratios of >10× the upper limit of normal (ULN) were only observed in 0.5% of cases. The clinical liver symptoms requiring hospitalization were relatively rare (23/100,000 patients or 0.023% of cases). No liver failure or death was observed. These and other results indicate that diclofenac rarely causes severe liver injury in humans [169].

The United States (U.S.) Drug Induced Liver Injury Network (DILIN) is a prospective registry of severe idiosyncratic drug hepatotoxicity. Schmeltzer et al. [126] reported a study on liver injury caused by NSAIDs in the U.S. The authors conclude that hepatocellular injury is the most common manifestation seen with NSAID toxicity and diclofenac is the most frequently implicated NSAID agent (16/30 cases). Bort et al. [170] analyzed acute diclofenac cytotoxicity on human and rat hepatocytes and hepatic cell lines (HepG2, FaO). Diclofenac impaired ATP synthesis by mitochondria and the authors suggested that toxicity might be related to drug metabolism because diclofenac was more cytotoxic to drug metabolizing cells (rat and human primary hepatocytes) than to non-metabolizing cell lines (HepG2, FaO). The toxic effect was reduced by the addition of cytochrome P450 inhibitors and in vitro cytotoxicity of diclofenac correlated well with the generation of two metabolites: 5-hydroxydiclofenac and *N*,5-dihydroxydiclofenac.

Gómez-Lechón et al. [85] also analyzed the generation of ROS and the apoptotic effect of diclofenac after exposure of human and rat hepatocytes to diclofenac. Antioxidants were able to prevent caspase-8 and -9 activation by diclofenac and maintain mitochondrial integrity. The authors concluded that the mitochondrial pathway of apoptosis is the only (or major) pathway involved in diclofenac-induced apoptosis and that the strongest apoptotic effect was produced by the metabolite 5-hydroxydiclofenac.

Other studies reported similar findings, such as diclofenac (metabolite)-induced ROS generation, lipid peroxidation, mitochondrial injury, ATP depletion, GSH depletion, lysosomal fragmentation and DNA fragmentation [171,172,173]. Many of these signs of toxicity were reversed by antioxidants, MPT pore sealing agents, lysosomotropic agents and inhibitors of cytochrome P450 isoenzymes. The final common pathway of all these events is the leakage of cytochrome c from the mitochondrial intermembrane space into the cytosol, resulting in the activation of caspases-9 and 3, mitochondrial/lysosomal cross-talk and apoptosis.

Yano et al. [169] investigated the immune response in diclofenac-induced idiosyncratic hepatotoxicity in mice. Gene expression of the main interleukins (ILs) and chemokines involved in the inflammatory response and the expression of helper T (Th) 17 cell-derived factors in the liver were significantly increased, as well as the levels of IL-17 in plasma. The results suggest at least a partial involvement of IL-17 in the development of diclofenac-induced liver injury since antagonizing IL-17 reduced toxicity. In addition, both gene expression and plasma levels of IL-1β were rapidly increased after diclofenac administration suggesting its involvement in the pathogenesis of diclofenac-induced hepatotoxicity.

Interaction between drug-induced toxicity pathways and the pro-inflammatory cytokine tumor necrosis factor alpha (TNFα) was investigated in HepG2 cells by Fredriksson et al. [174]. Transcriptomics of the stress response pathways initiated by diclofenac and carbamazepine, revealed the endoplasmic reticulum (ER) stress/translational initiation signaling and nuclear factor-erythroid 2 (NF-E2)-related factor 2 (Nrf2) antioxidant signaling as two important affected pathways. Inhibition of the Nrf2-dependent adaptive oxidative stress response enhanced drug/TNFα-induced cytotoxicity but did not affect C/EBP homologous protein (CHOP) expression. Both hepatotoxic drugs enhanced expression of the translational initiation factor EIF4A1, which was essential for CHOP expression and drug/TNFα-mediated cell killing. The authors conclude from their data that diclofenac initiates PERK-mediated CHOP signaling in an EIF4A1 dependent manner, thereby sensitizing the hepatocyte towards caspase-8-dependent TNFα-induced apoptosis.

#### 4.1.4. Naproxen

Naproxen is a propionic acid derivative NSAID and has been available as OTC medication since 1994. Naproxen is an analgesic, antipyretic and anti-inflammatory drug. It is a non-selective inhibitor of the enzymes COX-1 and COX-2 and decreases synthesis of prostaglandins, important mediators in inflammatory and pain pathways. Currently, more than 10 million prescriptions for naproxen are filed each year but these numbers do not include the large-scale OTC sales. Side effects of naproxen include dizziness, dyspepsia, nausea, and abdominal discomfort, but rarely liver injury. Naproxen is metabolized by the cytochrome P450 system and idiosyncratic liver injury may be due to a toxic metabolite although the mechanism has not been completely elucidated yet [175,176].

The absorption of naproxen is rapid and complete when given orally and the biotransformation of this drug includes demethylation and glucuronidation, as well as sulfate conjugation reactions. The metabolites are excreted in urine, with only a small proportion of the drug being eliminated unchanged [177]. In human liver, CYP2C9 and CYP1A2 are involved in naproxen metabolism [178,179] and subsequent glucuronidation takes place via UDP-glucuronosyltransferase (UGT2B7) [180]. There is some evidence that naproxen metabolism is related to hepatotoxicity: Yokoyama et al. [181] reported that naproxen induces lipid peroxidation in isolated rat hepatocytes, resulting in cell death and formation of high molecular weight protein aggregates in the hepatocytes. Oxidative stress was demonstrated by the formation of TBARS, a marker of lipid peroxidation. The increase of TBARS strongly correlated with the decrease of intracellular GSH. The authors concluded that ROS production and lipid peroxidation are induced by the metabolism of naproxen in rat primary hepatocytes.

A follow-up study confirmed the pivotal role of the GSH/GSSG (glutathione/glutathione disulfide) ratio in naproxen-induced toxicity. Increased GSSG levels preceded lipid peroxidation and LDH release [182]. Ji et al. [183] reported that ferrous iron release contributes to naproxen-induced microsomal lipid peroxidation and that naproxen and salicylic acid are not uncouplers of cytochrome P450. Naproxen toxicity and disposition were also investigated in the isolated perfused liver. Lo et al. [184] investigated the disposition of naproxen, its reactive acyl glucuronide metabolite (NAG) and a mixture of NAG rearrangement isomers (isoNAG) and concluded that covalent protein-adducts were formed in the liver, with isoNAG being the more important substrate for adduct formation. Using the same experimental model, Yokoyama et al. [185] demonstrated that naproxen increased liver damage (AST, ALT) and peroxidation (TBARS). GSSG and TBARS content were significantly increased in naproxen-perfused liver. In addition, the biliary excretion (clearance) of indocyanine green, a compound used for testing liver function, was decreased. The authors concluded that the biliary excretion system was disrupted due to naproxen-induced hepatic oxidative stress. Naproxen-induced hepatotoxicity and liver injury is very rare (≈1–3 per 100,000 users), although cases of acute hepatitis have been reported within six weeks after the start of naproxen intake. Once naproxen intake is terminated, biochemical markers of liver injury such as AST and ALT usually return to normal levels [186].

Andrejak et al. [175] described a patient using naproxen at 500 mg/day with nausea, abdominal pain, malaise, jaundice and increased AST, ALT and alkaline phosphatase levels. Histology showed moderate hepatocellular necrosis. After termination of naproxen, the patient recovered rapidly. Ali et al. [176] described a patient who developed jaundice and intractable pruritus shortly after taking naproxen. Histological analysis showed inflammatory infiltration and a progressive loss of the small interlobular bile ducts (ductopenia). The authors suggest that normalization of liver function and histology after termination of naproxen consumption may take up to 10 years.

#### 4.1.5. Ibuprofen

Ibuprofen was the first member of propionic acid derivatives to be introduced in 1969 as a better alternative to ASA. Ibuprofen is the most frequently prescribed NSAID and it is also an OTC drug. Ibuprofen has excellent analgesic and anti-inflammatory properties as well as antipyretic activity because it inhibits both cyclooxygenases (COX-1 and COX-2). Ibuprofen is frequently used to relief pain related to dysmenorrhea, headache, and osteoarthritis or rheumatoid arthritis. Adverse reactions to ibuprofen appear to be dose and duration dependent and the major adverse effects are related to the gastrointestinal tract, the kidneys and blood coagulation. In addition, ibuprofen may produce dizziness, dyspepsia, bronchospasm, and hypersensitivity reactions, but rarely causes clinically apparent and serious acute liver injury [43,187,188].

In fact, ibuprofen at OTC doses does not represent a risk for developing liver injury, because it has a short plasma half-life and it does not give rise to toxic metabolites (e.g., covalent modification of liver proteins by the quinine-imine metabolites of paracetamol or irreversible acetylation of biomolecules by ASA) [189]. High doses of ibuprofen (2400 to 3200 mg daily) may produce increased ALT plasma levels (<100 U/L), although clinically apparent liver injury due to ibuprofen is very rare. Only a few cases of ibuprofen-induced acute liver toxicity have been reported. The mechanism of toxicity has not been completely elucidated and may be multifactorial. Most of the ibuprofen-induced cases of hepatotoxicity have a rapid onset suggesting the production of a reactive metabolite as well as the involvement of a hypersensitivity response related to an immuno-allergic reaction [188]. Underlying liver diseases like hepatitis C may increase the risk of ibuprofen-induced acute liver injury [154,190]. Finally, Basturk et al. [191] published a case report of a seven-year-old patient who developed toxic epidermal necrolysis and vanishing bile duct syndrome (VBDS) after oral ibuprofen intake. Acute VBDS is a rare disease with unknown etiology. The patient was treated with supportive care (a steroid and ursodeoxycholic acid), with complete recovery after eight months.

## 5. Endogenous Antioxidant Defense Systems

Reactive Oxygen Species (ROS) are produced during normal intracellular metabolism and from exogenous substances. They play an important role in a range of biological processes, e.g., in the defense against microorganisms, as second messengers in several signaling pathways, and in modulating gene expression. Moreover, when generated in excess and when redox balance is disturbed, these free radicals can damage cellular organelles and induce inflammation, ischemia, apoptosis, and necrosis. Since living organisms are continuously exposed to free radicals, cells have developed antioxidant defense mechanisms. These antioxidant defense mechanisms include molecules and enzymes formed endogenously and bioactive molecules obtained from food (reviewed in [192]).

Any substance or compound that scavenges free radicals or non-radical ROS or RNS, or inhibits cellular oxidation reactions can be considered an antioxidant [193]. Endogenous antioxidants are capable to counteract the deleterious effects of free radicals and maintain cellular homeostasis. These endogenous antioxidants include both enzymatic antioxidants such as catalase, superoxide dismutases, glutathione peroxidases, peroxiredoxins, and thioredoxins [194] as well as non-enzymatic antioxidants (glutathione, urate, bilirubin, melatonin). The coordinated action of antioxidant enzymes ensures efficient ROS removal and promotes repair [195].

Free radicals, ROS and RNS are very reactive and since they are generated in different cell organelles, enzymatic defense systems have evolved in different cellular compartments. One of the most effective intracellular enzymatic antioxidants is superoxide dismutase (SOD) which catalyzes the dismutation of O_2_^•−^ (superoxide anions) to O_2_ and to the less-reactive species H_2_O_2_ with remarkably high reaction rates. This is accomplished by successive oxidation and reduction of the transition metal ions incorporated in the SOD enzymes (reviewed in [196]). The enzyme SOD is widespread in nature and present in all oxygen-metabolizing cells [197]. In humans, there are three superoxide dismutases: mitochondrial Mn-SOD, cytosolic Cu/Zn-SOD, and extracellular ecSOD. The main function of all SODs is to protect the cell from harmful effects of superoxide anions. In addition, extracellular SOD (ecSOD) is known to affect endothelial cells by preventing NO from reacting with superoxide anions (reviewed by [198]).

Glutathione peroxidase (GPx), catalase (CAT) and peroxiredoxins (Prx) control the ultimate fate of hydrogen peroxide produced from the superoxide anions by SODs.

Peroxisomes are the major storage site of catalase, an enzyme that catalyzes the biotransformation of H_2_O_2_ into water and O_2_. In addition, catalase has a peroxidative activity promoting the reaction between H_2_O_2_ and hydrogen donors to generate water and oxidize the reduced donor [199]. Although catalase might not be essential for all cell types, under normal conditions its deficiency in conditions of oxidative stress can increase cell damage and death [200].

GPx enzymes protect the cells against oxidative stress through the reduction of hydroperoxides using GSH as electron donor, yielding water and oxidized GSSG (glutathione disulfide). This antioxidant property is important for cellular homeostasis since hydroperoxides can be substrates for the Fenton reaction which induces oxidative stress through the production of the highly reactive hydroxyl radical. Five different isoenzymes of GPx have been identified but their expression depends on the tissue and species (reviewed in [200]).

Peroxiredoxins (Prx) are a group of 25-kDa proteins present in organisms of all kingdoms including humans. They contain a conserved cysteine (Cys) residue giving them the capacity to donate electrons and inactivate hydroperoxides and peroxynitrite. Six different isoforms of Prx (PrxI to PrxVI) have been identified in mammals, varying in number and position of the Cys residues. These enzymes may protect cellular components against hydroperoxides produced by normal metabolism and prevent cellular oxidative damage (reviewed in [201]).

The thioredoxin system is composed of thioredoxin (Trx) and thioredoxin reductases (TrxR), a group of enzymes that belong to the pyridine nucleotide-disulfide oxidoreductases family. The thioredoxin system plays an important role in DNA synthesis, defense against oxidative stress, redox signaling and apoptosis. Trx expression is very high in the intestine and has an important role in the gut immune response [202].

The tripeptide GSH (γ-glutamylcysteinylglycine) is synthetized in the cytosol and is an essential non-enzymatic regulator of intracellular redox homeostasis. The cysteine residue present in this compound allows GSH to inactivate oxidants through the reversible oxidation of its active thiol forming the oxidized form GSSG. GSH is a ubiquitous antioxidant present in many organelles and has two important functions: (1) to scavenge or inactivate ROS, RNS and electrophilic compounds generated in cellular metabolism; and (2) to function as a substrate for GPx to inactivate hydroperoxides and prevent the generation of hydroxyl radicals. Mitochondria contain large stores of GSH. Mitochondria are important organelles that produce a substantial amount of ROS. It has been reported that mitochondrial GSH (mGSH) has a vital role in maintaining the integrity and function of the mitochondria and its depletion may trigger undesirable events that promote mitochondrial dysfunction and cell death (reviewed in [203,204]).

Other important molecules in the cell that have antioxidant capacity include metal-binding proteins. The function of these proteins is to sequester metals such as iron and copper, preventing transition-metal catalyzed generation of radicals, e.g., transferrin and lactoferrin bind iron while albumin binds copper. Other molecules, such as bilirubin, melatonin, lipoic acid, coenzyme Q and uric acid, have also been proposed to act as antioxidants [192].

Protection of cellular systems against oxidative stress is also achieved via transcriptional regulation of antioxidant enzymes. Various transcription factors are involved in the regulation of the expression of antioxidant enzymes such as SOD, catalase, GPx, Prx and Trx. These transcription factors include Nrf1/2, PGC1-α and Foxo3a, often acting together, e.g., as co-activators [195,205,206].

The nuclear transcription factor E2-related factor 2 (Nrf2) belongs to the Cap‘n’Collar/basic leucine zipper (CNC-bZIP) family of proteins [207]. Nrf2 protein is normally inactive and located in the cytoplasm bound to its inhibitor Kelch-like ECH-associated protein 1 (Keap1) [208]. Nrf2 is an oxidative stress sensor which, once activated by oxidants, dissociates from Keap1 and translocates to the nucleus to induce transcription of target genes involved in the protection against oxidative stress (reviewed in [209]). Nrf2 has also been demonstrated to play a role in the protection against drug-related toxicity and xenobiotics-induced carcinogenesis via the induction of phase II enzymes such as UDP-glucuronosyltransferase (UGT), sulfotransferases (SULT), NAD(P)H:quinone oxidoreductase 1 (NQO1) and glutathione S-transferase (GST). These enzymes are involved in the metabolic inactivation and detoxification of drugs and carcinogens [210]. Nrf2 is also involved in the transcriptional induction of the antioxidant enzymes γ-glutamylcysteine synthetase (γ-GCS) and heme oxygenase-1 (HO-1) [211]. Nrf2 induces transcription via binding to the antioxidant response element (ARE) within the 5′-flanking region of its target genes [212].

**Figure 4:**
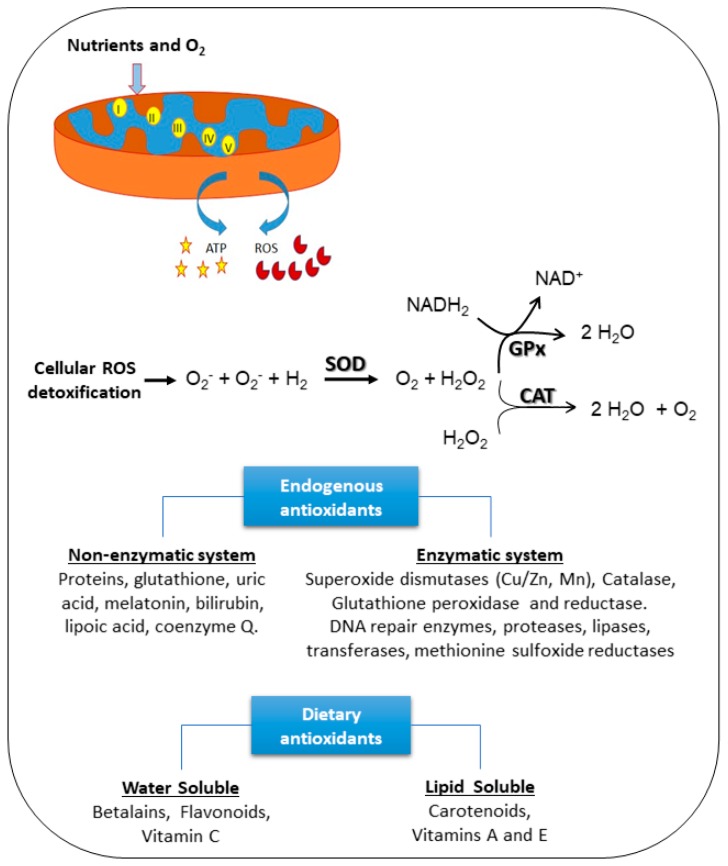
Cellular antioxidant defense systems. ROS, reactive oxygen species; SOD, superoxide dismutase.

The concerted actions of the enzymatic and non-enzymatic antioxidant defense systems are essential for the effective detoxification of ROS, RNS, electrophilic compounds and carcinogens and the maintenance of cellular redox homeostasis and cell survival (Figure 4).

This also implies that when endogenous antioxidant systems are compromised or exhausted, cells will no longer be able to cope with oxidative stress. This will ultimately result in cellular dysfunction and death. Therefore, it is important to obtain antioxidants from dietary intake to provide an extra line of defense against cellular oxidative stress. 

## 6. Dietary Natural Pigments with Biological Activity

Dietary natural products with therapeutic activity have been used for centuries for the treatment of human diseases. Vegetables and fruits are very important in human nutrition as sources of nutrients and phytochemicals that reduce the risk of several diseases [213] such as cancer (reviewed in [214]), cardiovascular disease [215], neurodegenerative diseases (reviewed in [216]), type 2 diabetes [217] and hypertension [218].

It is estimated that the frequent intake of vegetables and fruits might prevent up to one third of cancer-related deaths in the United States [219]. Several in vitro and animal studies suggest that the biological activities of vegetables, fruits and derivatives are frequently related to their antioxidant capacity (reviewed in [40,220]). Therefore, the health benefits of diets rich in vegetables, fruits and derived products such as beverages are due not only to fibers, vitamins and minerals, but also to a diversity of plant pigments and secondary metabolites with potential biological activity in humans (reviewed in [12]). Wang et al. reviewed different studies of natural extracts such as *Dunaliella salina*, *Hydnora africana*, *Calotropis procera*, *Zingiber officinale* Rosc, *Hibiscus sabdariffa* L., *Aloe barbadensis* Miller, *Lycium barbarum* and food-derived compounds such as resveratrol, apigenin, silymarin, lupeol, sesamol, ellagic acid, gallic acid, picroliv, curcumin, β-carotene, sulforaphane, and α-lipoic acid, against acetaminophen-induced hepatotoxicity. All of them showed a protective effect against the cellular oxidative stress induced by the highly reactive acetaminophen metabolite NAPQI but they appear to have different mechanisms of detoxification depending on the method of extraction, reagents and dosage used (reviewed in [40]). In addition, an in vitro study with 20 fruits commonly consumed in the American diet (apple, avocado, banana, blueberry, cantaloupe, cherry, cranberry, red and white grape, grapefruit, lemon, melon, nectarine, orange, peach, pear, pineapple, plum, strawberry, and watermelon) suggest that there is synergism between these compounds that improves the antioxidant capacity compared with single antioxidant compounds [221].

Vitamins, pigments and other phenolic compounds are widely distributed in the plant kingdom and can be found in vegetables, fruits, roots, seeds, leaves, and other natural products such as honey, propolis and royal jelly. Natural products show a vast diversity of phytochemical compounds, including carotenoids, betalains, flavonoids, and other phenolic compounds [222]. They include structurally simple molecules as well as complex oligo/polymeric assemblies with very specific bio-physicochemical properties making them important candidates for new drugs [223]. 

Colored compounds or pigments, are generated from natural sources such as plants, animals, fungi and microorganisms and serve vital functions in cellular processes such as photosynthesis, protection of cells against UV light and oxidative stress and transport of oxygen in blood (heme). Dietary natural pigments can be classified by their structural characteristics as benzopyran derivatives (e.g., flavonoids), *N*-heterocyclic compounds (e.g., betalains) and isoprenoid derivatives (e.g., carotenoids) (reviewed in [224]).

The presence or absence of many bioactive compounds in natural products depends on geographical and environmental factors, such as humidity, temperature, season, pollution and altitude, as well as conditions of growth and storage, and the presence of genetically different varieties [225]. Therefore, it is difficult to standardize the chemical composition of natural products, but it is possible to link their biological activity to the presence of specific compounds with demonstrated therapeutic activity [226].

Plant-derived antioxidants, in particular pigments, have gained considerable importance due to their health benefits. Recently, plant foods and food-derived antioxidants have received growing attention, because they are known to function as chemopreventive agents and to protect against oxidative damage and genotoxicity [227]. The National Research Council (NRC) has recommended eating five or more servings of fruits and vegetables to increase the health benefits [228]. 

In addition, there is still a lack of knowledge about the potential therapeutic effect of plant-derived compounds against OTC analgesic and NSAIDs-induced acute liver failure. Therefore, additional animal studies and clinical trials are needed to discover new antidotes and therapeutic doses from natural sources to prevent or reduce drug-induced liver toxicity and acute failure.

### 6.1. Flavonoids

Flavonoids belong to the class of plant phenolic pigments with low molecular weight with a characteristic flavan nucleus as their main structure. These plant components have a protective function against UV radiation, pathogens and herbivores. They are distributed throughout the entire plant and more than 10,000 different flavonoid compounds have been identified [229,230].

All green plant cells are capable of synthesizing flavonoids. They may participate in the light-dependent phase of photosynthesis to catalyze electron transport, regulating the ion channels involved in photophosphoregulation [231]. Biosynthesis invariably starts with the ubiquitous amino acid phenylalanine through the phenylpropanoid pathway and takes different pathways depending on the kind of flavonoid that is synthesized (reviewed in [232,233,234]). The chemical nature of flavonoids depends on their structural class, degree of hydroxylation, additional substitutions and conjugations, and degree of polymerization [235]. They frequently occur attached to sugars (glycosides) increasing their water solubility [236]. Humans and animals cannot produce these plant-based antioxidants and they are normally obtained through the dietary consumption of vegetables, fruits, and teas [237]. 

**Figure 5:**
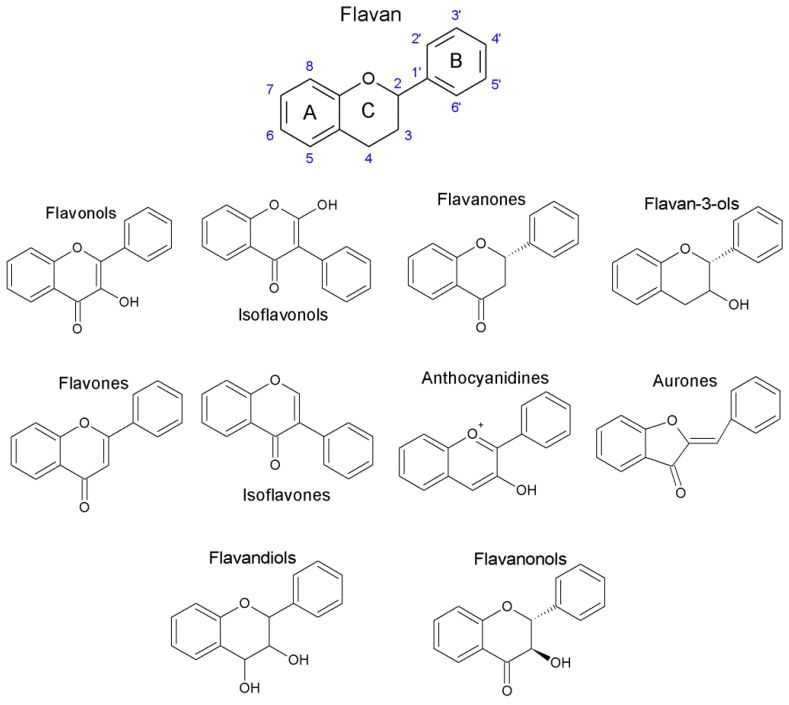
Basic flavonoid structure (flavan) and main classification of flavonoids.

Flavonoids are all derived from from a flavan nucleus composed of two phenolic rings (A and B) and one oxane (C) with 15 carbon atoms (C6-C3-C6) [238]. Flavonoids can be divided in different subclasses, based on the connecting position of the B and C rings as well as the degree of saturation, oxidation and hydroxylation of the C ring. These sub-classes include flavonols, isoflavonols, flavanones, flavan-3-ols (or catechins), flavones, isoflavones, anthocyanidins, aurones, flavandiols and flavanonols (Figure 5) (reviewed in [232,233,239]).

The total dietary intake of flavonoids such as flavanones, flavonols, flavones, anthocyanins, catechins, and biflavans was initially estimated to be up to 1 g/day in the United States [240]. Subsequent studies suggested that those amounts were somewhat overestimated and the daily human intake of flavonoids was re-estimated to be approximately 23 mg/day [241,242]. Human consumption of flavonoids is not limited to only plant foods [243] but also via intake of plant extracts such as tea, coffee, and red wine [244]. Bioavailability of flavonoids depends on the food source, dosage and their physicochemical properties such as molecular size, lipophilicity, solubility and pKa. In nature, flavonoids are usually bound to sugars such as β-glycosides except for flavan-3-ols and proanthocyanidins. Glycoside conjugates need to be hydrolyzed in the small intestine to release the aglycone flavonoid and be absorbed by passive diffusion through the epithelium. After absorption, flavonoids can react with oxidants to inactivate them or they are metabolized in the liver, e.g., via conjugation reactions. Therefore, aglycone flavonoids cannot be detected in urine or plasma (reviewed in [239]). Recent evidence suggests that the colon plays an important role in the bioavailability of flavonoids since analysis of urinary flavonoid metabolites indicate the presence of colonic metabolites. These results suggest that some of these compounds and their metabolites may play an important role in the protective properties of a vegetable and fruit-rich diet (reviewed in [12]). Flavonoids have a short half-life (≈2–3 h) and they do not accumulate in the body explaining their low toxicity even when ingested for prolonged periods. Despite this, flavonoids might interfere in the biotransformation of many commonly used drugs such as losartan, digoxin, cyclosporine, vinblastine and fexofenadine by the inhibition of CYP450 enzymes, thus increasing their pharmacological potency (reviewed in [232,245]).

Flavonoids have played a major role as medical treatment in ancient times and their use has continued to present day [232]. Flavonoids can inhibit several important enzymes including ATPase, aldose reductase, hexokinase and tyrosine kinase [246]. Flavonoids can also induce several enzymes, e.g., aryl hydroxylase and epoxide hydroxylase. Thus, flavonoids seem to possess several pharmacological properties that make them excellent agents to serve as natural biological response modifiers [231]. In addition, modulation of the activity of pro-inflammatory enzymes is one of the most important mechanisms of action for flavonoids. Pro-inflammatory enzymes, such as cytosolic phospholipase A2 (cPLA2), cyclooxygenases (COX), lipoxygenases (LOX), and inducible NO synthase (iNOS), produce very potent inflammatory mediators and therefore their inhibition contributes to the overall anti-inflammatory potential of flavonoids (reviewed in [247]).

The flavonoid-related antioxidant activity is mainly due to their free radical scavenging potential. The rate and efficiency of this biological activity is determined by the presence of structural features such as the number and position of hydroxyl groups (A, B and C rings) and 2,3-double bond conjugated with a 4-oxo function (C ring). These structural features facilitate electron delocalization and radical absorption [248]. A quantum chemical study of flavonoids explains that the 3-hydroxyl group in ring C plays an important role in the activity of flavonols due to its capacity to interact with the positions 2′ and 6′ of ring B and the 4-keto group of ring C, making them the most potent flavonoid antioxidants [249]. In addition, in vivo studies have demonstrated that flavonoids can also indirectly induce antioxidant enzymes and increase the concentration of uric acid in plasma (reviewed in [250]). Based on these structural characteristics and biological activities, flavonoids have been evaluated in various diseases and toxicity models, including liver injury and toxicity to determine their protective effect (reviewed in [239]). Quercetin, one of the most abundant flavonoids, has been tested in different models of liver toxicity including carbon tetrachloride, ethanol, clivorine, thioacetamide and paracetamol. In these studies, orally or intraperitoneally administered quercetin, at doses of 40–90 mg/kg for mice and 20–50 mg/kg for rat, showed a strong inhibition of oxidative stress-induced injury, enhancing the redox status and reducing inflammation [251,252,253,254,255]. A mixture of flavonoids from *German chamomile* (160 mg/kg p.o. (per os) in vivo, and 250–750 µg/mL in vitro) and apigenin-7-glucoside (AP7Glu) (10–60 µM in vitro) protected against ethanol- and CCl_4_-induced hepatocellular injury in rats and primary rat hepatocytes by normalizing ceramide content [256]. Another study showed that a mixture of nine flavonoids identified from *Laggera alata* extract had hepatoprotective effects at doses (orally) of 50–200 mg/kg in vivo and 1–100 µg/mL in vitro against CCl_4_-induced liver injury in rats and primary rat hepatocytes due to its potent antioxidative and anti-inflammatory activity. These results also suggest that flavonoids can scavenge reactive oxygen species by non-enzymatic mechanisms and enhance the activity of hepatic antioxidant enzymes [257]. High glucose-induced oxidative stress contributes diabetes-related liver pathology. Anthocyanins have been reported to reduce intracellular reactive oxygen species (ROS) levels and cyanidin-3-*O*-β-glucoside (C3G) had the highest antioxidant activity. This flavonoid contributed to the prevention of hyperglycemia-induced hepatic oxidative damage, both in HepG2 cells (1–100 µM) and in mice (100 mg/kg p.o.). This compound induced the synthesis of glutathione (GSH) via increasing the expression of the glutamate-cysteine ligase catalytic subunit (Gclc) gene through the activation of protein kinase A (PKA) and the phosphorylation of cAMP-response element binding protein (CREB) as the target transcription factor (independently of Nrf1/2 transcription factors) [258]. In addition, in a study of anti-retroviral drugs-induced oxidative stress and liver toxicity in rats, silibinin (100 mg/kg p.o.), one of the most well-known and potent hepatoprotective flavonolignanes isolated from *Silybum marianum*, improved all biochemical and pathological parameters, confirming its hepatoprotective and antioxidant potential [259]. These studies suggest that dietary intake of flavonoids contributes to the prevention or treatment of hepatotoxicity induced by the exposure to xenobiotics and other environmental factors.

### 6.2. Betalains

**Figure 6:**
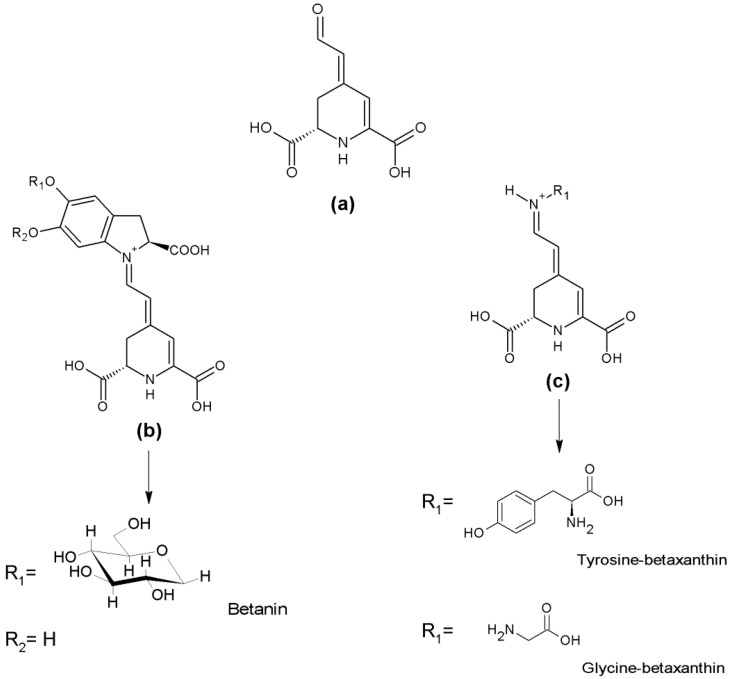
Betalamic acid (**a**), precursor of betalains. Betacyanins (**b**) and betaxanthins (**c**), as the main classes with some derivatives.

Betalains are water-soluble plant pigments that contain nitrogen. They provide protection against UV radiation and pathogens and can act as optical attractants to pollinators. They are synthesized from the amino acid tyrosine by the condensation of betalamic acid, which is a common chromophore of all betalains. Betalains can be classified into betacyanins (red-violet) or betaxanthins (yellow-orange) depending on the nature of the groups conjugated to the betalamic acid (Figure 6). Betacyanins (e.g., betanin and betanidin) and betaxanthins (e.g., vulgaxanthin I and II) are divided into several subclasses, based on the chemical characteristics of the betalamic acid conjugate [260].

Betalamic acid can be conjugated with cyclo-3,4-dihydroxyphenylalanine (*cyclo*-Dopa) to form betacyanins and with amino acids or amines to form betaxanthins. The groups conjugated to the betalamic acid determine the absorption wavelength of around 480 nm for betaxanthins and around 540 nm for betacyanins. Structural modifications and the presence of side chains (e.g., sugars and amino acids) cause hypso- or bathochromic shifts (shorter or longer) in the absorption wavelengths [260,261,262].

Betalain pigments are particularly abundant in the Caryophyllales order and can be found in roots, flowers, fruits and some vegetative tissues of plants [263]. However, betalains are not produced in the Caryophyllaceae and Molluginaceae families, since these families accumulate anthocyanins, flavonoid-derived pigments. Betalains and anthocyanins have never been reported together in the same plant, suggesting they are mutually exclusive [264]. This appears to be an evolutionary mechanism of adaptation to protect against environmental factors and predators [265]. 

Betalains are cationic compounds with a high affinity for negatively charged membranes, thus improving their efficacy as antioxidants. The active cyclic amine group of betalains functions as hydrogen donor and confers reducing properties to these compounds. In addition, the betacyanins such as betanin and betanidin have an enhanced antioxidant capacity in vitro compared to betaxanthins, catechin and α-tocopherol. This is due to the presence of a phenolic ring which increases their electron transfer capability, making them superior antioxidants [266]. It has been suggested that the free radical scavenging activity of betanin is pH-dependent. It is also important to consider the contribution of anionic forms of cyclo-DOPA-5-*O*-β-d-glucoside to the antioxidant activity of betanin at basic pH. These data suggest that betanin is a better hydrogen and electron donor at higher pH, contributing to its enhanced free radical-scavenging activity [267]. 

Several studies have demonstrated potent radical scavenging activity of betalains and their derivatives in vitro [268,269,270,271,272]. The antioxidant protection against reactive radical species may be mediated either by direct free radical scavenging or by the induction of endogenous antioxidant defense mechanisms under the control of redox-regulated transcription factors such as Nrf2 [266]. Nrf2 orchestrates the expression of genes encoding antioxidant and phase II enzymes such as heme-oxygenase 1 (HO-1), NAD(P)H:quinone oxidoreductase 1 (NQO1) and glutathione S-transferases (GST). The chemo-preventive effect of betanin is due to the induction of endogenous redox-system enzymes and glutathione synthesis mediated by Nrf2 dependent signal transduction pathways [272]. Chemopreventive compounds may enhance the transcriptional activity of Nrf2 independently from Keap1 [273].

The dietary intake of betalains in humans is limited, since they can be obtained only from red beet, swiss chard, amaranthus, cactus pear, pitaya and some tubers and their derived products. They are also used as food colorants providing another dietary source of these phytochemicals. The intake of red beet juice or cactus pear revealed the bioavailability of betalains. They can be absorbed by the small intestine into the systemic circulation in their intact forms, indicating that hydrolysis reactions are not always necessary for absorption as reported for some glycosylated flavonoids, although the bioavailability of betalains has been reported to be only ≈1% of the consumed amount. The maximum concentration of betalains is detected in plasma 3 h after intake. Moreover, some unknown metabolites have been detected in urine after betalain intake. The biological actions of these metabolites are unknown. Betalains are capable to bind to biological membranes reducing their bioavailability and capacity to react with other macromolecules. The major commercially exploited source of betalain is red beetroot (*Beta vulgaris*), which contains two major soluble pigments: betanin (red) and vulgaxanthine I (yellow). Previous studies reported that the betacyanin and betaxanthin content of red beet roots varies between 0.04–0.21% and 0.02–0.14%, respectively, depending on the cultivar, although some new varieties have higher betalain content (reviewed in [274]). Red beet roots contain a large amount of betanin, 300–600 mg/kg, and lower concentrations of isobetanin, betanidin and betaxanthins [266]. The prickly pear (*Opuntia ficus indica*) contains about 50 mg/kg of betanin and 26 mg/kg of indicaxanthin [269]. *Opuntia robusta* and *Opuntia streptacantha* fruit juices also contain large amounts of betacyanins (333 and 87 mg/L, respectively) and betaxanthin (134 and 36 mg/L, respectively) [7]. The concentration of betacyanins in red pitaya (*Hylocereus* cacti), expressed as betanin equivalents ranges from 100–400 mg/kg depending on the species [275,276].

Thanks to the increasing interest in the antioxidant properties of betalains, some studies have focused on their benefits against oxidative stress-related organ damage, although there are only a few studies on the hepatoprotective effect of betalains. Red beet juice and other red beet products, frequently used in dietary products, contain many bioactive compounds, the most important being betalains and may provide protection against certain oxidative stress-related disorders in humans [266]. Betalains from the berries of *Rivina humilis* showed dose-dependent cytotoxicity (0–40 µg/mL) in HepG2 hepatoma cells [277]. Frequent consumption of purple *Opuntia* cactus fruit juices rich in betalains protects hepatocytes against oxidative stress and improves the redox balance in acetaminophen-induced acute liver toxicity in vivo (800 mg/kg of lyophilized juice p.o.) and in vitro (8% *v*/*v*) [7]. In vitro studies have subsequently demonstrated that the free radical scavenging capacity of betanin (1–35 µM) via activation of the Nrf2 pathway and subsequent induction of Nrf2 target genes, is responsible for its hepatoprotective and anticarcinogenic effects [272,273]. Betanin (1–4% in fodder) also attenuated CCl_4_-induced liver damage in common carp (*Cyprinus carpio* L.) by the inhibition of CYP2E1 activity and reduction of oxidative stress [278]. The role of betalains as hepatoprotective and chemopreventive compounds can be summarized by their ability to stabilize cellular membranes, scavenge free radicals or electrophilic metabolites, and improve the cellular redox-balance. Nevertheless, the optimal daily intake of betalains to achieve hepatoprotection in humans has not been elucidated yet due to their apparent poor stability and bioavailability. In addition, there is still a lack of information regarding the safety of consumption of processed betalains and their possible pharmacological interactions, necessitating more toxicological and clinical studies.

### 6.3. Carotenoids

Carotenoids are colored (red, orange, and yellow) pigments widely distributed in nature with over 700 structurally different compounds [279]. These lipophilic molecules are synthesized in photosynthetic organelles as well as in fruits, flowers, seeds, and storage roots. Carotenoids have important functions in photosynthesis in plant and algae, protection against UV light and in photomorphogenesis. Some microorganisms such as yeast and bacteria can also produce carotenoids that function in alleviating photo-oxidative damage [280,281]. Structurally, carotenoids are composed of eight isoprenoid units and can be considered lycopene (C_40_H_56_) derivatives. The synthesis of this group of pigments involves hydrogenation, dehydrogenation, cyclization, oxidation, double bond migration, methyl migration, chain elongation or chain shortening [282].

**Figure 7:**
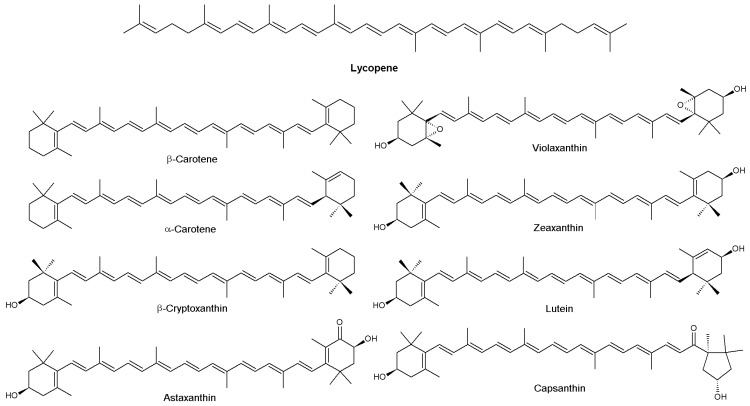
Chemical structure of lycopene with the main primary and secondary carotenoids.

The natural functions and actions of carotenoids are determined by their physical and chemical properties, which are defined by the molecular structure. Carotenoids must have a specific molecular geometry to ensure their biological activity. The presence and number of double bonds in their structures define to a large extent the chemical properties, e.g., lipophilicity that facilitates their ability to inactivate free radicals and protect cellular membranes [283]. The carotenoid classification is based on their chemical composition: carotenoids composed of carbon and hydrogen and carotenoids composed of carbon, hydrogen and oxygen, also called oxycarotenoids or xanthophylls. Alternatively, carotenoids may also be classified as primary carotenoids that are required for plant photosynthesis (β-carotene, violaxanthin, and neoxanthin) and secondary carotenoids that serve as UV protectors and attractants present in flowers and fruits (α-carotene, β-cryptoxanthin, zeaxanthin, lutein, astaxanthin, and capsanthin) [284] (Figure 7).

Carotenoid biosynthesis in plants is highly regulated, although all processes involved are not completely elucidated yet. In recent decades, most carotenogenic genes from various plants, yeasts and algae have been identified and their functions elucidated [281]. Carotenoids are synthesized via the isoprenoid pathway [285] in a highly regulated process using isopentenyl pyrophosphate (IPP) as a common precursor of many isoprenoid compounds. The synthesis and amount of these pigments in plants are based on the requirements to maintain vital functions and to protect them against environmental factors [286]. Humans and animals depend on dietary intake for carotenoid supply, since they cannot synthesize carotenoids de novo [287].

Humans only have access to 40–50 of all structurally identified carotenoids via the diet, although only ≈20 have been detected in human blood plasma including α-, β- and γ-carotene, lycopene, α- and β-cryptoxanthin, zeaxanthin, lutein, neurosporene, phytofluene and phytoene (reviewed in [288]). These carotenoids are abundant in tomatoes, carrots, oranges, beans, beet, broccoli, Brussel sprouts, coleslaw, celery, zucchini, pepper, spinach, cucumber, mango, and watermelon [289]. Recommended dietary intake is based on the contribution of carotenoids to (pro)vitamin A content. Vitamin A (retinol) can be obtained preformed from the diet through meat and dairy products, or from vegetables and fruits as carotenoids that are subsequently converted into provitamin A. It has been estimated that 14 µg of β-carotene is necessary to yield 1 µg of retinol (i.e., 1 retinol activity equivalent). Based on FAO and WHO recommendations, an intake of 700–900 µg of retinol per day is recommended, equal to approximately 11 mg/day of β-carotene [290]. It has been suggested that only ≈5% of the total intake of carotenoids is absorbed in the small intestine. Once absorbed, carotenoids can be detected in plasma lipoproteins, mainly LDL. Carotenoid absorption, bioavailability, breakdown, transport and storage is dependent on a number of factors, including the type of carotenoid and dietary source, as well as genetic factors, nutritional status, age, gender and diseases. Moreover, it has been reported that individual carotenoids may inhibit the absorption of each other. In addition, carotenoid availability may be decreased by interactions with ASA and sulfonamides (reviewed in [280,288]). Carotenoids are also found in nutritional supplements and other dietary sources since they are also used as food colorants [291], since they have been increasingly exploited by food, nutraceutical and pharmaceutical companies due to the recent interest in their biological potential as antioxidants and treatment against chronic or age-related diseases in humans [280].

The direct antioxidant activity of carotenoids, both in vitro and in vivo, depends on the presence and number of functional groups such as carbonyl and hydroxyl groups. Thus, capsanthin and astaxanthin display better antioxidant activity than β-carotene or zeaxanthin. Besides, carotenoids appear to modulate the expression of antioxidant enzymes and it has been suggested that carotenoids might have a synergistic effect with other dietary antioxidants (reviewed in [292]). The antioxidant action of carotenoids is due to their ability to inactivate singlet oxygen and other free radicals. Moreover, carotenoids preferentially quench peroxyl radicals even in the presence of other oxidizable substrates. They also act as chain-breaking antioxidants, thus preventing lipid peroxidation and protecting cell membranes, with an almost equally potency as α-tocopherol [293].

Carotenoids accumulate mainly in the liver and are incorporated into lipoproteins for release into the circulation. Carotenoids contribute to the antioxidant defense system in the liver as scavengers of free radicals. In patients with chronic liver diseases, micronutrient antioxidants are severely depleted in serum and liver tissue and liver injury is associated with decreased antioxidant levels, particularly carotenoids. Carotenoids may have beneficial effect in non-alcoholic fatty liver disease (NAFLD) probably via multiple mechanisms, including antioxidant and anti-inflammatory effects and regulation of M1/M2 macrophage polarization (reviewed in [294]).

Carotenoids also demonstrate benefits in cancer and stroke since in these diseases free radicals play an important role. It has been suggested that carotenoids influence the strength and fluidity of membranes, thus affecting its permeability to oxygen and other molecules. In vivo and in vitro studies have shown that the photo-protective role of carotenoids is related to its antioxidant capacity (reviewed in [283,295]).

Recent studies have demonstrated the hepatoprotective action of carotenoids in different models of liver toxicity and hepatitis. Bixin, the main carotenoid from *Bixa Orellana* L. (annatto) seeds, used prophylactically for seven days at 5 mg/kg p.o. protected against CCl_4_-induced liver toxicity by reducing lipid peroxidation [296]. In the same model of liver toxicity, similar results were obtained with a prophylactic treatment for 14 days with astaxanthin (ASX) and astaxanthin esters (ASXEs) at 100 and 250 µg/kg p.o. from the green microalga *Haematococcus pluvialis* [297]. Likewise, lycopene-enriched tomato paste (at lycopene equivalence doses of 0.5 and 2.5 mg/kg p.o. for 28 days prior intoxication) increased the activity of cellular antioxidant enzymes such as superoxide dismutase, catalase and GSH-peroxidase and reduced microsomal lipid peroxidation induced by *N*-nitrosodiethylamine in rat liver [298]. In a rat high fat diet model of NAFLD, lycopene at 5, 10 and 20 mg/kg p.o. for six weeks, reduced liver damage, increased both enzymatic and non-enzymatic antioxidant defenses and reduced levels of the pro-inflammatory cytokine TNFα [299]. Prophylactic treatment of lycopene at 10 mg/kg i.p. (intraperitoneally) for six days was also protective against d-galactosamine/Lipopolysaccharide-induced hepatitis in rats [300]. These studies demonstrate that carotenoids are potent antioxidants and protect cellular membranes, suggesting that they can also be protective against oxidative liver damage induced by acute and chronic exposure to analgesic and non-steroidal anti-inflammatory OTC drugs.

## 7. Concluding Remarks

The overconsumption and misuse of OTC analgesic drugs is a clinical problem of epidemic proportion. It is the cause of a significant rise in acute and chronic liver diseases, especially in developing countries, due to a lack of information about the risk of self-medication and a lack of affordable access to general practitioners. Plant and natural products as traditional medicine have been used for many centuries throughout the world. Currently, there is a trend towards increased consumption of these products to treat or prevent many diseases. It has been demonstrated that many of these products and their secondary metabolites have beneficial effects in many different pathologies, especially those associated with inflammation and oxidative stress. An important issue that still needs attention is the bioavailability of natural pigments. For example, there is still considerable controversy about the bioavailability of hydrophilic flavonoids and betalains, in contrast to, e.g., carotenoids. Therefore, additional information about the pharmacokinetic and pharmacodynamic properties of these compounds are needed. Natural pigments appear to be biologically active at very low concentrations and their effects go beyond that of simple anti-oxidants. Natural pigments, or their metabolites, often induce highly specific intracellular responses modulating specific signaling pathways to protect cells. These specific qualities make natural dietary pigments excellent candidates for the treatment and/or prevention of OTC-drug-induced liver diseases. Several studies have evaluated the use of crude extracts or isolated compounds of natural products and observed beneficial effects in cell culture or animal models. However, these studies were mostly performed using the natural pigments as prophylactic. The challenges for the future application of these products as therapeutic or preventive interventions are: (1) identifying the most effective compounds in crude extracts; (2) to reconstitute the most effective mixtures of purified compounds that preserve synergy between individual components; (3) the reproducible cultivation and preparation of (extracts of) natural products with adequate bioanalytical characterization and quality control; and (4) pre-clinical studies in humans to extend the experimental findings to clinical application.

## Figures and Tables

**Table 1:** Range of therapeutic dosage per day of acetaminophen and non-steroidal anti-inflammatory drugs (NSAIDs) in humans and current treatments against intoxications.

Drug	Therapeutic Dosage in Adults (Orally) per Day	Mechanism of Toxicity	Treatment or Antidote
Acetaminophen (Paracetamol, APAP, Tylenol^®^, Johnson & Johnson, New Brunswick, NJ, USA)	325–4000 mg/day	*N*-acetyl-*p*-benzoquinone imine (NAPQI)-induced mitochondrial dysfunction and oxidative stress	*N*-acetylcysteine (NAC) 70–140 mg/kg, and activated charcoal to reduce the absorption of the drug
Acetylsalicylic acid (ASA, Aspirin^®^, Bayer AG, Leverkusen, Germany)	500–4000 mg/day	Mitochondrial dysfunction and oxidative stress induced by salicylic acid and its oxidated metabolite gentisic acid	Gastric lavage and sodium bicarbonate perfusion to reduce acidity and increase excretion of salicylic acid
Diclofenac (Cataflam^®^, Novartis AG, East Hanover, NJ, USA)	50–200 mg/day	Thiol-reactive quinone imines-induced mitochondrial dysfunction and oxidative stress	Diuresis and dialysis to enhance the excretion of the drug
Naproxen (Aleve^®^, Bayer AG, Leverkusen, Germany)	220–660 mg/day	Metabolite-induced oxidative stress and liver damage	Gastric lavage and activated charcoal to reduce the absorption of the drug
Ibuprofen (Advil^®^, Pfizer Inc., New York, NY, USA)	200–1200 mg/day	Hypersensitivity response related to an immuno-allergic reaction	Gastric lavage and activated charcoal to reduce the absorption of the drug
